# The Effect of Baseline on Toddler Event-Related Mu-Rhythm Modulation

**DOI:** 10.3390/brainsci11091159

**Published:** 2021-08-31

**Authors:** Caterina Piazza, Eleonora Visintin, Gianluigi Reni, Rosario Montirosso

**Affiliations:** 1 Bioengineering Laboratory, Scientific Institute, IRCCS E. Medea, Bosisio Parini, 23842 Lecco, Italy; gianluigi.reni@lanostrafamiglia.it; 2 0-3 Center for the At-Risk Infants, Scientific Institute, IRCCS E. Medea, Bosisio Parini, 23842 Lecco, Italy; eleonora.visintin@lanostrafamiglia.it (E.V.); rosario.montirosso@lanostrafamiglia.it (R.M.)

**Keywords:** baseline, mu-rhythm, EEG, ERD/ERS, pediatric population

## Abstract

Event-related mu-rhythm activity has become a common tool for the investigation of different socio-cognitive processes in pediatric populations. The estimation of the mu-rhythm desynchronization/synchronization (mu-ERD/ERS) in a specific task is usually computed in relation to a baseline condition. In the present study, we investigated the effect that different types of baseline might have on toddler mu-ERD/ERS related to an action observation (AO) and action execution (AE) task. Specifically, we compared mu-ERD/ERS values computed using as a baseline: (1) the observation of a static image (BL1) and (2) a period of stillness (BL2). Our results showed that the majority of the subjects suppressed the mu-rhythm in response to the task and presented a greater mu-ERD for one of the two baselines. In some cases, one of the two baselines was not even able to produce a significant mu-ERD, and the preferred baseline varied among subjects even if most of them were more sensitive to the BL1, thus suggesting that this could be a good baseline to elicit mu-rhythm modulations in toddlers. These results recommended some considerations for the design and analysis of mu-rhythm studies involving pediatric subjects: in particular, the importance of verifying the mu-rhythm activity during baseline, the relevance of single-subject analysis, the possibility of including more than one baseline condition, and caution in the choice of the baseline and in the interpretation of the results of studies investigating mu-rhythm activity in pediatric populations.

## 1. Introduction

Electrophysiological (EEG) mu-rhythm has been increasingly studied in the last decade by developmental cognitive neuroscientists due to its association with the activity of the human mirror neuron system and its potentiality in investigating different cognitive processes (e.g., imitation, action interpretation, language, touch perception) that are crucial for the development of many social-cognitive skills [1,2,3,4,5,6,7,8]. This makes the mu-rhythm an interesting tool for the study of both healthy and clinical pediatric populations, underlying the neural correlates of the development of such skills and potentially identifying possible biomarkers of an atypical development [9,10,11], which can be used for improving the diagnosis processes and designing new rehabilitation programs.

In this framework, mu-rhythm is typically investigated by computing the event-related desynchronization/synchronization (ERD/ERS), which indicates the modulation of EEG mu-rhythm in response to a specific experimental event. This entails the collection of a baseline measure of the EEG mu-rhythm that ideally allows quantifying the variation in the mu-rhythm activity elicited by the analyzed event. Specifically, ERD/ERS is usually computed in terms of percentage change of EEG-mu-rhythm power between the baseline condition and the experimental event: ((event mu-power—baseline mu-power)/baseline mu-power) × 100 [12]. Therefore, negative values represent a mu-rhythm desynchronization, and positive values represent a mu-rhythm synchronization, and zero means no change between the baseline activity and the event. Since this equation is a simple ratio, it is easy to understand that ERD/ERS values depend on the magnitude of the mu-power recorded during the baseline condition. Thus, the choice of an adequate baseline measure is something to be carefully evaluated when designing an experiment aimed at studying the mu-rhythm activity [13] since this choice might even affect whether the mu-rhythm ERD/ERS is found in a particular experiment.

A wide variety of baseline conditions were used in both pediatric and adult studies, such as static or moving images, moving objects, static or moving body parts and periods of stillness with the absence of stimuli (e.g., [9,14,15,16,17,18,19,20,21]). However, the impact of different types of baselines on the study of mu-rhythm modulation has been scarcely investigated in the literature. We found only one study that systematically compared the effect of different baselines on healthy adult mu-ERD/ERS [22]. Specifically, Tangwiriyasakul and colleagues used five different baseline movies (two static, one quasi-static and two dynamic) in a motion imagery task and showed that the majority of the subjects had significantly higher mu-power for particular baseline conditions, thus resulting in more pronounced mu-ERD. However, the optimal baseline was subject-dependent, and they did not find a common preferred baseline. The remaining subjects were divided into three groups: “non mu-suppressive”, subjects that did not suppress their mu-rhythm during the task, “mu-absent”, subjects that did not show a mu peak in the power spectra related to the considered baselines and “no preference baseline”, subjects that showed a similar mu-rhythm desynchronization with all the analyzed baselines. The study confirmed the strong dependence on the baseline of the analyzed mu-rhythm modulation and highlighted a high inter-subject variability.

This suggests that it is necessary to be careful in interpreting results related to the mu-rhythm activity. As previously stated, the chosen baseline might affect the identification or not of task-related mu-rhythm modulations, but it might also strongly affect group analysis results if single subjects differently respond to the baseline condition and finally, it makes tricky the comparison among different studies.

To the best of our knowledge, there are no studies involving pediatric populations that investigated the effects of the baseline on mu-rhythm activity. Moreover, it should be noted that baseline measures collected in infants and toddlers also present methodological challenges [13]. For example, it is not easy to keep the toddler attentive looking at static images or without the presence of any stimuli. On the contrary, moving stimuli might be more attractive, but they might elicit desynchronization due to the higher engagement, thus affecting the comparison with a test condition.

In the present study, we explored the effect of two different baseline measures on mu-rhythm suppression in response to the observation and execution of goal-directed action in a group of healthy toddlers. Action observation (AO) and action execution (AE) paradigms are among the most used for the investigation of the mu-rhythm in pediatric populations addressing various topics. Lepage and Theoret [23] examined mu-rhythm activity during the execution and observation of a precision grip, as well as the observation of a non-goal-directed movement in 4–11-year-old children. They found desynchronization to the execution and the observation of the goal-directed action (grip) but not to the non-goal-directed one. Similar results were found in younger populations. Southgate and colleagues [24] identified mu-rhythm desynchronization in 9-month-old infants while they grasped a toy and while they observed a grasping action, but no desynchronization was found while infants were observing a non-goal-directed action. Nyström et al. [21] replicated the result in 8-month-old infants, but they did not include in the protocol the action execution condition. Marshall and colleagues [14] found desynchronization to both execution and observation of goal-directed action (button press) in 14-month-old infants, and the same action was also found to desynchronize the mu-rhythm during both observations and execution in pre-term infants [9]. Other studies used AO/AE protocols to investigate if the experience is related to the mu-rhythm activity. Saby et al. [6] found the same desynchronization in response to the execution of different goal-directed actions in 12-month-old infants, but the mu attenuation during the observation was higher when observing an action that matched the most recently executed one. Reid et al. [19] found higher mu-rhythm desynchronization in 14-month-old infants when observing action within their motor repertoire in respect to action non in the repertoire. However, Virji-Babul and colleagues [25] found similar desynchronization to action within and not within the motor repertoire in 4–11-month-old infants, and Stapel et al. [26] reported higher mu-reactivity to extraordinary actions than usual actions in 12-month-old infants. Unfortunately, none of these studies were included in the protocol action execution trials, thus lacking important information. More recently, Bryant and Cuevas [27] used an AO/AE task to evaluate the effects of observational (i.e., visual experience) and active training (i.e., motor experience) on mu/alpha-rhythm activity in 3–6-year-old children. They found that only occipital alpha rhythm was sensitive to the type of experience. Finally, Filippi and colleagues [4] investigated the association between motor system activation and social behavior in 7-month-old infants by means of an AO/AE protocol, and the results supported the hypothesis that mu-rhythm activation predicts other individuals’ goals. Despite the relatively good amount of studies investigating the mu-rhythm response to AO and AE in infancy, results are not always consistent, and the comparison between different studies is complicated due to the different ways used to investigate the mu-rhythm activity, the different baseline conditions used and the different scalp sites analyzed. As previously pointed out, we think that baseline measures can strongly affect the mu-ERD/ERS values, and we would like to systematically investigate the effect of two different baselines on mu-reactivity to AO and AE.

We selected two types of baselines among the ones previously used in AO/AE studies with toddlers or infants. Specifically, the first baseline condition we used was a short period of stillness in which no stimuli were presented. This choice was made in order to use a baseline the more natural as possible, which was designed to reproduce a condition identical to the experimental set-up except for the variable of interest. This condition was suggested to be an “optimal” baseline by Cuevas and colleagues [13], who wrote best practices for the collection of infant EEG mu-rhythm. The main issues associated with this baseline might be related to the difficulty in keeping the toddlers calm and attentive without the presentation of any stimuli and to the fact that this completely free condition might induce very different reactions in the participants resulting in different EEG states. The other used baseline consisted of the presentation on a screen of a black and white static image. The idea was to use a more controlled baseline condition and, at the same time, not to introduce a stimulus that might be too engaging and might affect the mu-rhythm activity.

The aims of the study were: (i) to verify if the two baselines were adequate to detect the mu-rhythm ERD; (ii) to assess if different ERD values resulted from the two baselines and identify a possible optimal baseline among the two proposed ones.

## 2. Material and Methods

### 2.1. Participants

A group of 42 typically developing toddlers were involved in the protocol. Inclusion criteria were: full-term infants (gestational age ≥ 37 weeks and birth weight ≥ 2500 g); no congenital abnormalities and uncomplicated prenatal, perinatal and neonatal courses; no developmental delay; and no motor or sensory deficits. Among the recruited subjects, 3 were excluded because of technical problems; 10 did not tolerate the EEG cap; 2 were distressed during the task, and the procedure was interrupted; 8 did not have a sufficient amount of good EEG data in at least one of the procedure conditions. Thus, 19 subjects were finally included in the present study (11 males and 9 females; age in months mean (M) = 33.42, standard deviation (SD) = 13.57, range = 14.7–59.1).

The study was conducted according to the guidelines of the Declaration of Helsinki and approved by the Ethics Committee of the Scientific Institute IRCCS E. Medea (Bosisio Parini, Lecco, Italy). Written informed consent to participate was obtained from all parents prior to inclusion in the protocol.

### 2.2. EEG Data Recording and Experimental Task

EEG signals were recorded using a dense-array EGI system (Electric Geodesic, Inc., Eugene, OR, USA) equipped with 60/64-electrode caps (HydroCel Geodesic Sensor net). Data were referenced to the vertex, sampled at 250 Hz and bandpass filtered between 0.1–100 Hz.

**Figure 1:**
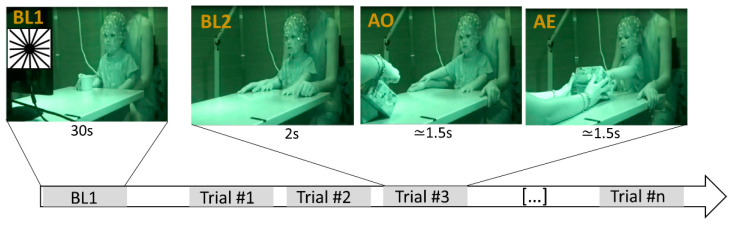
Experimental procedure. The procedure started with BL1, during which a static black and white image (reported in the figure in the top left corner) was presented for 30 s on a screen positioned in front of the participant. Successively, the toddler completed a series of identical trials, each comprising 3 events: BL2, in which the toddler quietly looked at the experimenter for 2 s; AO, in which the toddler observed the experimenter performing the button press action; AE, in which the toddler performed the button press action.

The acquisition took place in a sound-attenuated and electrically shielded room while the toddler sat at a table on their parent’s lap. The experimental procedure consisted of the observation (action observation (AO) condition) and the execution (action execution (AE) condition) of a button press action [9]. Both AO and AE were performed using a custom-made wood box with a recessed button. Two different baseline conditions were used: (1) the presentation of a static black and white image (Baseline 1, BL1); (2) short periods of stillness executed at the beginning of each trial (Baseline 2, BL2) (Figure 1). The procedure started with the presentation of the black and white static image on a screen positioned in front of the toddler (BL1). The image presentation lasted 30 s. After BL1, the experimenter sat in front of the toddler and started a series of trials, each comprising 3 epochs: BL2, AO and AE (Figure 1). During BL2, the experimenter hid the button-box under the table for 2 s, and no stimuli were presented to the toddler, who was expected to be quiet, looking at the experimenter. Successively, the experimenter brought out the button-box and performed the action of pressing the button with the right index finger while the toddler was looking at him. Finally, during the AE condition, the experimenter handed out the box to the toddler, who was expected to imitate the button press action. The experiment continued as long as the toddler remained collaborative, interested and tolerated the EEG cap. Thus, the number of completed trials varied from toddler to toddler; on average, 23.5 (SD = 4.3, range = 14–30) complete trials were performed.

The task was videotaped with a digital camera (Sony HDR-HC9, HDV 1080i, Sony Europe B.V., Weybridge, Surrey UK) connected to the EEG system so that the video was synchronous with the EEG recorded data. The video recording was analyzed offline to identify and reject trial epochs in which the toddler was not behaving according to the task and to check the hand that they used to perform the button press action. The last information was used to compute a lateralized manipulation score for each toddler that allows the evaluation of the hand preference [9,28]. The score is calculated as follows: (%Right − %Left/%Right + %Left) and it results in a value between 1 and −1, where positive values indicate a right preference in hand use, whereas negative values indicate a left preference. Only 7 subjects always performed the button press action with the same hand (2 toddlers used the left hand and 5 the right hand). Among the other subjects, 6 had a right hand preference, 4 had a left hand preference and 2 subjects showed no preference (i.e., pressed the button exactly half time with the right hand and half with the left hand; Table 1).

### 2.3. EEG Data Processing

Raw EEG data were exported in a Matlab-compatible format (The Mathworks, Natick, MA, USA) and processed within the open-source EEGLAB signal processing environment [29] and custom Matlab scripts.

Continuous EEG data were filtered with a 1 Hz high pass and a 45 Hz low pass finite impulse response (FIR) filter. The clean_rawdata EEGLAB plug-in was used to identify flat channels (i.e., channels with flat line duration > 5 s) and channels poorly correlated with their interpolated reconstruction based on neighboring channels (correlation threshold = 0.85). After a visual inspection, the identified channels were interpolated with a spherical spline. No more than 15% of channels out of 60 were interpolated (M = 4.8, SD = 2.4, range = 1–10). The same plug-in was used to reject data periods in which more than 50% of the channels were contaminated by artifacts. The EEG signals were then re-referenced to average reference and segmented according to the behavioral events, which were automatically triggered. Specifically, the whole BL1 period of 30 s, BL2 epochs of 2000 ms and AO/AE epochs of 1500 ms were considered. For the latter, we used the 1500 ms prior to the experimenter or infant button press, which is approximately the timing from the beginning of the arm movement to action culmination [9,24]. Epochs containing artifacts were identified and rejected using an automatic algorithm (i.e., abnormal amplitude test with threshold 200 μV) [29], followed by visual inspection. Moreover, the video recording was used to reject a portion of BL1 data in which the toddler was not looking at the image, BL2 epochs in which the toddler was not in a resting state and AO epochs in which the toddler was not paying attention to the experimenter gesture, performed gross motor movements or movements similar to a reaching, pointing or button-pressing. As a result, the following data were used for the subsequent analysis: a mean of 20.8 s of BL1 continuous data (SD = 9.2, range = 6.2–30) and a mean of 11.7 BL2 (SD = 3.9, range = 5–18), 15.8 AO (SD = 5.5, range = 5–25) and 13.6 AE (SD = 5.8, range = 6–24) epochs.

The power spectral density (PSD) was estimated for each epoch using Welch’s method (Hamming window of 250 samples and 125 samples overlap) and successively log-transformed PSD = log10(1 + PSD).

Given the wide age range of the participants involved in the present study, as well as the lack of consensus in the mu frequency band for infants and toddlers [13], personalized mu frequency bands were used. To determine the band of interest, we calculated each toddler’s maximally attenuated frequency during the AE phase in respect to the two baseline conditions, and in both cases, we selected a 3 Hz band centered on this frequency. The identification of the maximally attenuated frequency was performed using a broad frequency band (4–13 Hz) and averaging the identified value across channels [9,30]. To ensure the weak stationarity of the analyzed epochs, an outlier analysis was performed on PSD values in each epoch. No outliers were identified.

Finally, event-related changes in band power between BL1, BL2 and AO, AE epochs were calculated as follows: ERD\ERS = [(A-R)/R] × 100 [12] where A denotes the power during the AO or AE epochs and R denotes the average power during the baseline.

**Figure 2:**
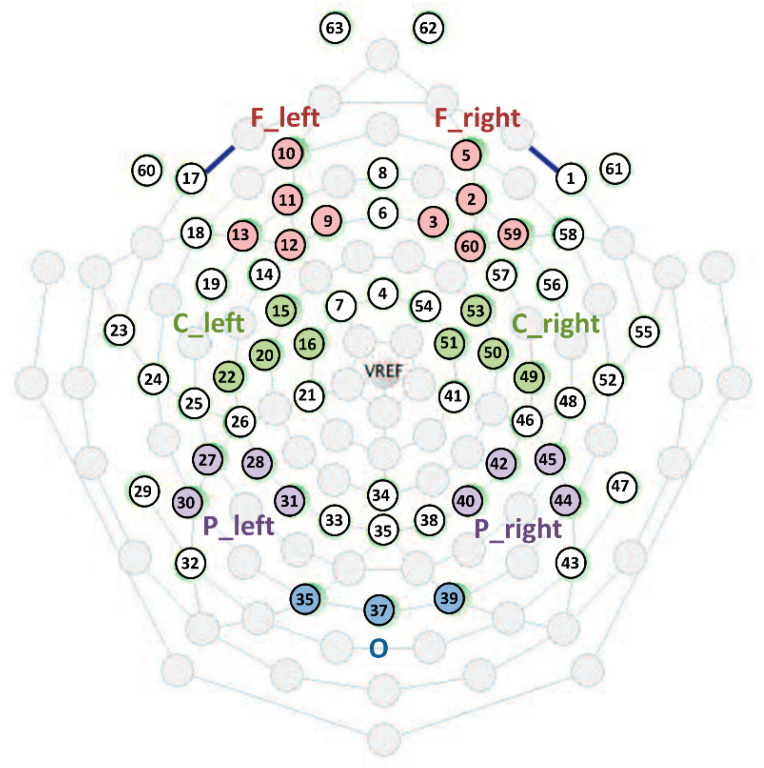
Sensor layout and representation of the created clusters of electrodes.

Event-related power changes were analyzed in multiple locations across the scalp topography [9]. Seven clusters were created by averaging the single-channel ERD/ERS values: frontal left (F_left), frontal right (F_right), central left (C_left), central right, (C_right), parietal left (P_left), parietal right (P_right) and occipital (O) (Figure 2).

### 2.4. Statistical Analysis

For each subject, significant mu-rhythm desynchronizations during AE were identified in each cluster by contrasting ERD/ERS values against a null hypothesis of zero median (i.e., no power changes between baseline and AE) using a series of one-sample Wilcoxon signed rank tests. The false coverage statement rate (FCR) adjustment [31] was applied to correct for multiple comparisons. Successively, for the subjects that showed significant mu-rhythm desynchronizations, a series of paired Wilcoxon tests were applied to test for significant differences between the ERD/ERS values computed using the two different baselines. Again, the FCR adjustment was applied to correct for multiple comparisons. The same analyses were repeated for the AO condition. All statistical analyses were performed at a significant level *p* < 0.050 using Matlab (The Mathworks, Natick, MA, USA).

## 3. Results

### 3.1. Identification of Mu-Rhythm Suppression

The mean frequency with the maximal PSD suppression during AE was 7.0 Hz (SD = 1.0, range = 5–8) if calculated in respect to BL1 and 6.7 Hz (SD = 0.9, range = 5–8) in respect to BL2 with no statistical differences between the two conditions (*p* = 0.331, paired *t*-test). Thus, the analyzed frequency range varied from 4–6 Hz to 7–9 Hz in both cases.

**Figure 3:**
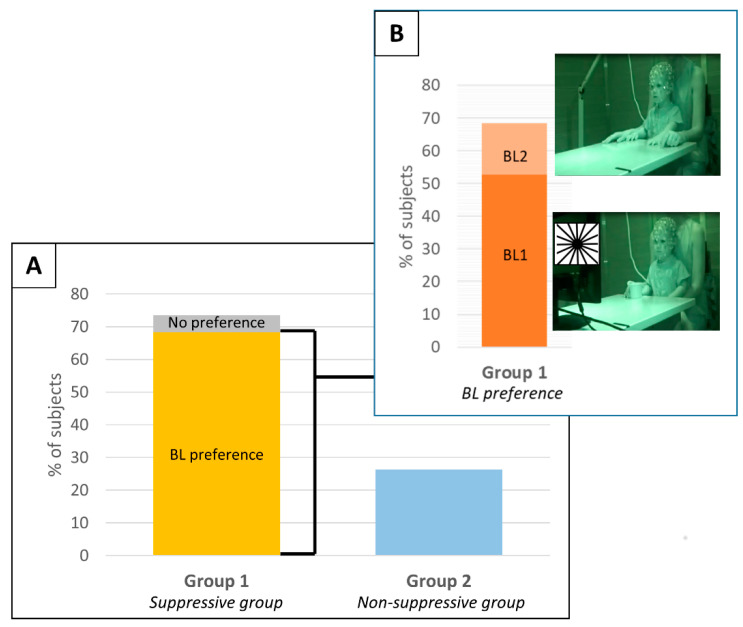
(Panel **A**) shows the percentage of subjects in the two identified groups: Group 1, *suppressive group*, and Group 2, *Non-suppressive group*. The subjects of Group 1 are divided between the ones that showed a baseline preference (yellow) and the ones that did not have a baseline preference (gray). (Panel **B**) showed, among the subjects of group 1 that had a baseline preference, the ones that preferred BL1 (dark orange) and the ones that preferred BL2 (light orange).

**Figure 4:**
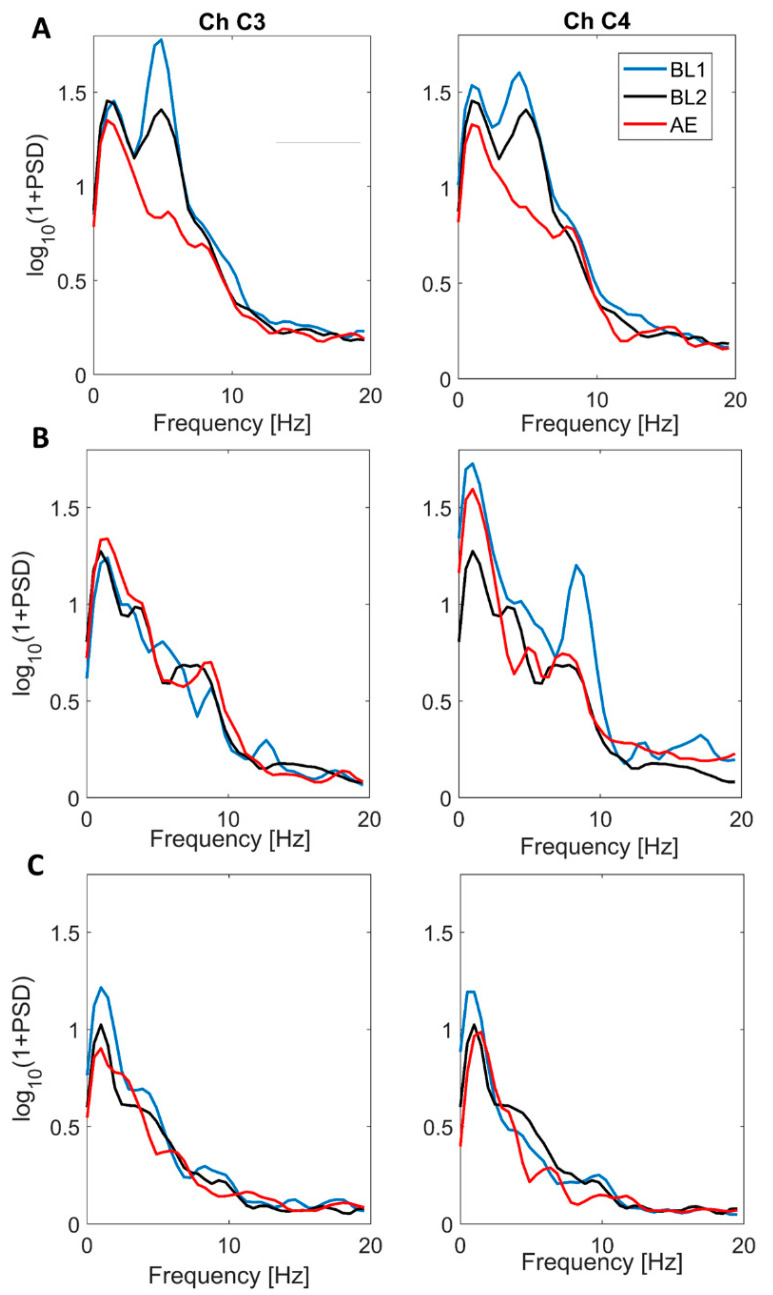
Power spectral density (PSD) estimated on two representative channels of the central clusters (channel 3, left column and channel 4, right column) during BL1 (blu), BL2 (black) and AE (red) for one subject of the Group 1 (Panel **A**), one subject of Group 2 showing a mu-rhythm peak (Panel **B**) and one subject of Group 2 that doesn’t have a clear mu-rhythm peak during baseline conditions (Panel **C**).

AE single subject ERD/ERS values are reported in Table 2. Subjects were divided into two groups according to the identification of a significant mu-rhythm suppression during the execution of the button press action (Figure 3). Group 1: suppressive group, i.e., subjects with a significant mu-rhythm suppression during AE trials in respect to at least one baseline condition (14 subjects, 73.7%; nine males and five females; age in months: median = 33.83, IQR = 18.9, range = 19.4–53.8). Group 2: non-suppressive group, i.e., subjects without a significant mu-rhythm ERD during AE trials (five subjects, 26.3%; two males and three females; age in months median = 20.00, IQR = 9.8, range = 14.7–59.2). No statistical difference was found between the subject ages in the two groups (*p* = 0.219, Wilcoxon rank sum test). Among the subjects of Group 2, two did not show a clear baseline mu-power peak, whereas the other three showed a clear peak at least for one of the two baseline conditions but without a significant desynchronization (Figure 4). It should be noted that the lack of a significant desynchronization could also be attributed to the low number of AE epochs available for these subjects. Indeed, the number of AE epochs is significantly lower in Group 2 (Median = 8.0; IQR = 2.3) in respect to Group 1 (Median = 15.5; IQR = 9.0) (*p* = 0.001, Wilcoxon rank sum test). The members of the Group 2 were discarded from further analysis.

### 3.2. Baseline Preference

For each subject, the baseline that allows the identification of higher ERD during AE was considered as the optimal baseline. Thirteen subjects out of 14 showed significant ERD/ERS differences between the two baselines in at least one cluster (Figure 3, Table 2). Specifically, 10 subjects (71.4%) showed higher ERD values with BL1, 3 subjects (21.4%) showed higher ERD values with BL2 and only 1 subject (SBJ5) (7.1%) did not present a baseline preference (Figure 3).

To check the consistency of the baseline preference, the same analysis performed on AE epochs was repeated, taking into account AO epochs. The results of these analyses are reported in Table 3 and suggested that the baseline selected to be the preferred one during AE was responsible for a stronger mu-rhythm desynchronization during AO as well. Specifically, nine subjects (64.3%) confirmed their baseline preference, SBJ5, that did not show a preference in AE resulted in being more sensitive to BL2, and for the remaining four subjects, we did not identify significant mu-rhythm desynchronization during AO. However, qualitatively comparing the ERD/ERS values computed with the two baselines, the preference showed in AE seemed to be confirmed (Table 3).

**Figure 5:**
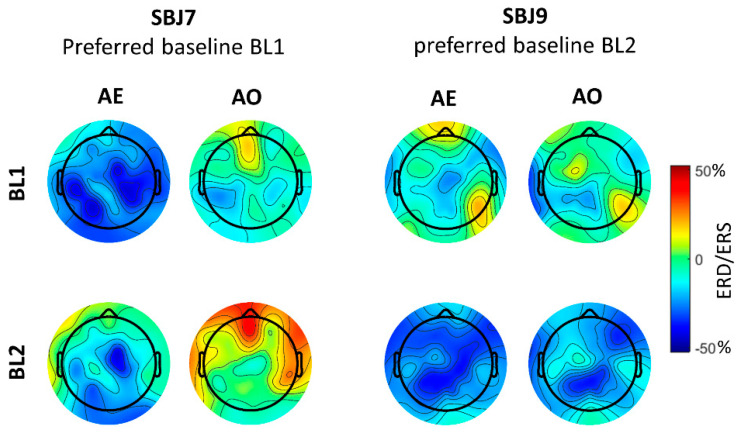
Topographic maps of mu-rhythm ERD/ERS [%] for SBJ7 and SBJ9, which show a preference for BL1 and BL2, respectively.

In Figure 4 we reported topographical maps of ERD/ERS values computed during AO and AE using both BL1 and BL2 for two subjects with different baseline preferences that were selected as typical examples. SBJ7 preferred BL1, for which he presented significant desynchronization for both AE and AO conditions in the majority of the analyzed scalp sites (Figure 5, Table 2 and Table 3). The desynchronization was more pronounced for the AE condition, as previously reported in the literature [9,23]. Looking at the mu-ERD/ERS computed using BL2 power value, we can identify desynchronization only during AE, especially in central, parietal and occipital areas (Figure 5), even though mu-ERD values resulted in being not significant (Table 2). Practically, no desynchronization was found for the AO condition (Figure 5, Table 3). On the other hand, SBJ9, who preferred BL2, showed clear and diffused mu-ERD for both AE and AO when using BL2 power value in the ERD/ERS calculation and presented only weak, not significant desynchronization in some scalp sites when taking into account BL1 power value (Figure 4, Table 2 and Table 3).

## 4. Discussion

In the present study, we investigated the relevance of the choice of the baseline measure used for the computation of mu-rhythm modulation in a pediatric population. This was performed by comparing mu-ERD/ERS values computed by using two different baseline conditions in an AO/AE task performed by 19 toddlers.

First of all, we identified the frequency band of interest by selecting the most attenuated frequency during AE. The selection was made using a broad frequency band (4–13 Hz), and this choice was based on the wide age range of the participants in the protocol and on the existing literature (see [13] for a review). The frequency identified for both BL1 and BL2 (7 Hz and 6.7 Hz, respectively) was in line with the values reported in the pediatric literature [13,30], and, as expected, there were no differences in the selected frequency between BL1 and BL2. Thus, the type of baseline does not affect the frequency band of the mu-rhythm.

The main findings of our work are reported in Figure 3, which shows how the baseline measure affects the mu-desynchronization in our sample of toddlers and are discussed below.

Specifically, the AE condition was analyzed in order to identify subjects that showed a significant desynchronization. As previously reported in the adult literature [22], the majority of the subjects (73.7%, Group 1) showed a significant mu-ERD during AE, at least in respect to one of the used baselines (Figure 3). However, it should be pointed out that only nine subjects (47.4%) showed a significant desynchronization with both the baselines (Table 2); thus, mu-ERD would not have been found for some subjects if using only one baseline condition with obvious effects in the investigation of the mu-rhythm modulations evoked by the task. This supports the importance of the baseline measure in allowing the study of the mu-rhythm modulation. The remaining subjects (26.3%, Group 2) did not have a significant mu-ERD (Figure 3), and this finding was also similar to what was previously found in adult subjects [22]. Since some of the toddlers in Group 2 showed a clear mu-rhythm peak in the PSD of at least one of the two considered baselines and some not, we think that the reasons why a significant mu-ERD was not found might be various. Specifically, we hypothesize that the lack of desynchronization might be attributed to: (1) the low number of AE epochs available; (2) the high intra-individual variability that characterizes infant and toddler neural responses [31]; (3) an unrelaxed condition of the subjects during the baselines that might have altered the resting mu-rhythm activity.

All the subjects of Group 1 (*suppressive group*), except one, showed a baseline preference during AE identified by means of a significantly greater mu-ERD (Table 2, Figure 3). The optimal baseline differed among subjects, and this finding is also in line with the adult literature [22]. However, if Tangwiriyasakul and colleagues [22] in their study did not identify a common preferred baseline, we found that a higher number of toddlers (10 subjects out of 13) preferred the BL1, static image baseline. This seems to be in contrast with the suggestion by Cuevas and colleagues [13] to consider a condition identical to the experimental condition except for the variable of interest as an optimal baseline. Though, this suggestion was not based on empirical data but on the idea to control for the possibility that the mu-rhythm desynchronization is activated only by a change in the type of stimuli between the baseline and the test. Some previous studies involving pediatric populations used a similar approach in selecting the baseline reporting mu-rhythm desynchronization. Nevertheless, the chosen baselines were very different. For example, [18] used as baseline moving objects that moved in a non-goal-directed manner, while in the test condition, they performed goal-directed movements. On the other hand, Nystrom and colleagues [21] used a baseline similar to our BL2, in which the experimenter sat passively, in contrast to the test conditions when he showed the infant different actions. In our sample, a more controlled baseline, i.e., a static image (BL1), resulted in enhancing mu-rhythm desynchronization for the majority of the subjects, thus representing a possible optimal baseline.

The result of the baseline preference was confirmed by the analysis of the AO condition. Indeed, for all the Group 1 subjects, the optimal baseline identified with AE analysis was also optimal for AO. However, not all the subjects showed a significant mu-ERD during AO. This was not so surprising since previous studies identified greater desynchronization during AE than AO (e.g., [9,18,19,23]). Moreover, as in the case of the absence of a significant mu-ERD during AE in the subject of Group 2, we have to take into account the high intra-individual variability of infant/toddler EEG [32].

Some limitations in the current study should be pointed out. First of all, the sample size is relatively small and characterized by a wide age range. Thus, future studies with larger samples and better-defined age ranges should be conducted for the generalization of our results. Secondly, only two types of baseline conditions were tested, and in particular, a dynamic baseline condition (i.e., moving shapes) was not included in the study. This is a strong limitation for the characterization of an optimal baseline. However, infant and toddler studies present some methodological challenges, including the difficulty in conducting a long experiment with multiple conditions and, at the same time, the necessity to perform as much repetition of the task as possible in order to obtain a reliable response [33]. Thus, we decided not to introduce more than two different baseline conditions, and we choose to use a more natural baseline as possible (BL2) and a more controlled baseline as possible (BL1).

## 5. Conclusions

The present study represents a first attempt to characterize the effect of different baseline measures on the estimation of the event-related mu suppression in a pediatric population. To our knowledge, this is the only study in the literature that addressed this issue. Even though the two baselines we used seemed to be adequate to detect mu-rhythm modulations, our results support the importance of the baseline in the characterization of the mu-ERD outcome, and the main findings of our study are reported in Table 4. Mu-ERD in a task might be elicited by a specific baseline and not by others with differences among subjects. For our protocol, it seems that a static image condition could be a good baseline for studying the mu-ERD in the majority of the subjects. Nevertheless, a universal optimal baseline probably does not exist, and in light of our results, we think there are some considerations to take into account when implementing a mu-rhythm study with pediatric populations. First, it could be a good idea to inspect single-subject spectra related to the chosen baseline verifying the presence or not of a mu-rhythm peak, and also to perform single-subject analysis in order to exclude from group analysis the subjects that are not sensitive to a specific type of baseline. Secondly, as already suggested in the best practice described by Cuevas and colleagues [13], we proved that it might be useful to include in the experimental protocol more than one baseline condition in order to assure the identification of the mu-ERD. Finally, much attention should be given to the comparison of the results coming from studies that used different types of baseline.

## Figures and Tables

**Table 1:** Toddler hand preference. Number of toddlers with right and left hand preference with the respective median values, interquartile ranges (IQR) and minimum/maximum values (Range) of the Lateralized manipulation score.

	N	Median	IQR	Range
Right preference	13	0.89	0.49	1–0.29
Left preference	4	−0.91	0.32	−0.3–−1
No preference	2	0	0	0

**Table 2:** The table shows, for each subject, the number of AE epochs analyzed, AE ERD/ERS values (median (IQR)) for each channel cluster and for the two baselines (BL1, BL2), the results of the paired Wilcoxon test comparing AE ERD/ERS computed with BL1 and BL2 (FCR adjusted *p*-value), the baseline preference. * denotes the significant mu-rhythm desynchronization (One simple Wilcoxon signed rank test, FCR adjusted *p*-value < 0.050). Results are divided for Group 1, *suppressive group*, and Group 2, *non-suppressive group*.

	N. AE Epochs	F_Left	F_Right	C_Left	C_Right	P_Left	P_Right	O	BL Preference
**GROUP 1**									
**SBJ2**	24								
ERD/ERS BL1		−30.8 (18.4) *	−9.3 (35.1)	−25.7 (37.5) *	−11.3 (36.0)	−8.8 (27.9)	−38.2 (14.3) *	−21.8 (20.6) *	
ERD/ERS BL2		−23.1 (30.3) *	−15.3 (39.1)	−13.5 (30.5)	−17.6 (20.8)	−13.2 (20.0) *	−16.0 (18.6) *	−19.2 (24.5) *	
Wilcoxon test adj *p*		**0.005**	0.319	**0.005**	0.245	0.080	**<0.001**	0.313	BL1
**SBJ3**	23								
ERD/ERS BL1		−47.8 (12.5) *	−48.8 (13.3) *	−46.3 (16.7) *	−43.6 (15.5) *	−46.9 (6.3) *	−53.5 (13.2) *	−37.4 (18.1) *	
ERD/ERS BL2		−34.5 (15.6) *	−36.0 (16.5) *	−35.7 (20.0) *	−33.6 (18.1) *	−36.2 (7.6) *	−44.3 (16.1) *	−26.5 (21.0) *	
Wilcoxon test adj *p*		**<0.001**	**<0.001**	**<0.001**	**<0.001**	**<0.001**	**<0.001**	**<0.001**	BL1
**SBJ5**	11								
ERD/ERS BL1		−12.1 (20.6)	−25.5 (32.9) *	−25.5 (44.4) *	−36.4 (19.1) *	−48.6 (17.1) *	−26.8 (29.8) *	−15.1 (17.7)	
ERD/ERS BL2		−13.7 (18.5) *	−30.2 (19.8) *	−5.1 (11.1)	−24.8 (18.6) *	−37.1 (18.1) *	−39.1 (16.3) *	−20.8 (19.9) *	
Wilcoxon test adj *p*		>0.999	>0.999	0.611	>0.999	>0.999	0.336	>0.999	No preference
**SBJ6**	21								
ERD/ERS BL1		−40.0 (14.9) *	−28.6 (21.6) *	−20.8 (24.7) *	−32.8 (23.9) *	−35.2 (11.2) *	−24.9 (22.1) *	−31.1 (32.5) *	
ERD/ERS BL2		−32.0 (19.6) *	−27.6 (20.7) *	−19.7 (14.5) *	−16.2 (25.2) *	−34.3 (16.8) *	−29.4 (25.6) *	−37.2 (34.5) *	
Wilcoxon test adj *p*		**0.008**	>0.999	>0.999	**0.003**	>0.999	>0.999	0.071	BL1
**SBJ7**	9								
ERD/ERS BL1		−27.6 (37.4)	−26.3 (6.7) *	−30.0 (31.7) *	−45.6 (16.8) *	−34.5 (12.9) *	−34.0 (24.1) *	−31.0 (9.9) *	
ERD/ERS BL2		−16.3 (43.4)	−12.7 (7.8)	−18.6 (36.8)	−35.9 (21.8)	−19.7 (15.7)	−20.6 (29.0)	−27.1 (10.2)	
Wilcoxon test adj *p*		**0.010**	**0.010**	**0.010**	**0.010**	**0.010**	**0.010**	**0.010**	BL1
**SBJ8**	18								
ERD/ERS BL1		−32.9 (30.6) *	−27.5 (20.1) *	−27.4 (29.3) *	−33.7 (7.6) *	−32.1 (21.0) *	−32.2 (17.6) *	−25.1 (17.4) *	
ERD/ERS BL2		−15.4 (29.9)	−19.9 (23.0) *	−6.4 (29.7)	−19.2 (20.9) *	−16.2 (23.0) *	−16.0 (13.8) *	−16.5 (12.1) *	
Wilcoxon test adj *p*		**0.001**	0.644	**0.001**	**0.010**	**0.003**	**0.001**	**0.002**	BL1
**SBJ9**	13								
ERD/ERS BL1		−6.8 (19.0)	−9.5 (31.2)	−6.4 (28.9)	−13.8 (25.6)	−4.7 (23.8)	10.9 (20.5)	−10.6 (15.3)	
ERD/ERS BL2		−29.8 (17.5) *	−29.6 (13.1) *	−23.9 (15.7) *	−36.4 (24.6) *	−32.3 (18.5) *	−29.6 (25.2) *	−20.2 (16.7) *	
Wilcoxon test adj *p*		**0.002**	**0.001**	0.174	**0.038**	**0.006**	**0.001**	0.208	BL2
**SBJ11**	20								
ERD/ERS BL1		−36.8 (29.8) *	−40.1 (23.1) *	−33.0 (29.1) *	−36.3 (37.7) *	−41.8 (18.5) *	−42.5 (23.1) *	−31.7 (14.4) *	
ERD/ERS BL2		−16.0 (24.8) *	−18.9 (24.7) *	−13.8 (32.2)	−10.3 (26.4)	−14.7 (24.2) *	−18.3 (19.9) *	−15.3 (18.6) *	
Wilcoxon test adj *p*		**0.002**	**0.002**	**0.007**	**0.002**	**0.002**	**0.002**	**0.029**	BL1
**SBJ12**	19								
ERD/ERS BL1		−28.6 (23.8) *	−17.7 (26.7) *	−24.9 (11.5) *	−18.0 (20.5) *	−18.5 (13.1) *	−34.3 (24.0) *	−10.5 (31.6)	
ERD/ERS BL2		−33.7 (21.8) *	−31.8 (22.2) *	−28.4 (11.2) *	−25.3 (19.5) *	−28.8 (11.8) *	−42.9 (21.8) *	−30.1 (24.4) *	
Wilcoxon test adj *p*		**<0.001**	**<0.001**	**<0.001**	**<0.001**	**<0.001**	**<0.001**	**<0.001**	BL2
**SBJ13**	18								
ERD/ERS BL1		−23.1 (20.2) *	−26.6 (22.8) *	−6.3 (31.8)	−16.3 (19.7) *	−26.5 (22.4) *	−12.3 (28.7) *	−27.8 (21.1) *	
ERD/ERS BL2		−15.8 (31.4) *	−10.2 (25.3) *	−19.3 (23.7) *	−14.3 (20.4) *	−16.1 (19.5) *	−5.8 (29.8)	−13.6 (18.9)	
Wilcoxon test adj *p*		>0.999	**0.026**	**0.029**	0.752	**0.006**	**0.072**	**0.026**	BL1
**SBJ14**	13								
ERD/ERS BL1		−14.0 (27.8) *	−14.9 (44.9)	−4.2 (21.7)	−17.7 (27.7)	−24.0 (23.1) *	−23.5 (13.8)	−25.2 (24.1) *	
ERD/ERS BL2		−5.9 (29.6)	−8.1 (47.9)	4.7 (23.9)	−14.8 (29.9)	−19.7 (24.4)	−19.1 (14.9)	−11.2 (28.7)	
Wilcoxon test adj *p*		**0.001**	**0.001**	**0.001**	**0.001**	**0.001**	**0.001**	**0.001**	BL1
**SBJ17**	10								
ERD/ERS BL1		−37.3 (27.8)	−26.9 (33.1)	−37.9 (18.8) *	−12.6 (22.1) *	−27.7 (34.5)	−33.7 (13.5) *	−19.5 (33.7) *	
ERD/ERS BL2		−45.9 (24.2) *	−37.1 (28.9) *	−45.1 (16.2) *	−23.7 (19.2) *	−32.2 (32.9)	−38.1 (12.6) *	−14.7 (35.1)	
Wilcoxon test adj *p*		**0.005**	**0.005**	**0.005**	**0.005**	**0.005**	**0.005**	**0.005**	BL2
**SBJ18**	11								
ERD/ERS BL1		−9.1 (38.7)	−23.4 (23.4) *	−30.8 (16.3) *	−38.4 (14.3) *	−36.7 (40.1)	−40.7 (14.1) *	−36.8 (14.9) *	
ERD/ERS BL2		−12.0 (24.3)	−18.4 (18.7)	−2.9 (24.3)	−18.7 (17.2)	−21.3 (26.3)	−12.1 (8.5)	−17.8 (20.9)	
Wilcoxon test adj *p*		0.947	0.251	**0.004**	**0.004**	0.088	**0.004**	**0.004**	BL1
**SBJ19**	10								
ERD/ERS BL1		−21.0 (26.9) *	−33.1 (21.0) *	−21.0 (19.9)	−31.0 (18.9) *	−35.7 (12.1) *	−31.6 (27.6) *	−18.5 (29.1)	
ERD/ERS BL2		−18.3 (24.5)	−16.4 (29.7)	−14.5 (15.0)	−5.9 (23.9)	−19.9 (15.5)	−21.2 (13.6)	−18.0 (30.4)	
Wilcoxon test adj *p*		0.234	0.222	0.484	0.124	**0.035**	0.222	>0.999	BL1
**GROUP 2**									
**SBJ1**	9								
ERD/ERS BL1		−36.1 (29.5)	−15.8 (10.3)	−11.1 (17.8)	−21.9 (64.2)	−27.6 (29.3)	−37.6 (31.8)	−32.4 (9.2)	
ERD/ERS BL2		−33.1 (30.7)	4.1 (13.4)	−10.7 (17.7)	16.9 (97.0)	−16.7 (32.0)	−18.7 (42.1)	−9.8 (12.1)	
**SBJ4**	6								
ERD/ERS BL1		−16.0 (27.2)	−13.1 (49.8)	−2.7 (36.1)	−13.9 (22.7)	−14.3 (15.5)	−14.1 (30.2)	10.5 (37.6)	
ERD/ERS BL2		−5.2 (30.7)	−18.2 (46.8)	−12.5 (32.2)	−1.6 (26.0)	−24.6 (13.8)	−16.6 (30.2)	7.8 (37.1)	
**SBJ10**	8								
ERD/ERS BL1		−38.7 (17.8)	−13.9 (16.5)	−6.0 (21.2)	−23.8 (19.7)	−25.4 (12.9)	−19.1 (30.3)	−7.2 (27.3)	
ERD/ERS BL2		−17.1 (22.4)	−10.4 (17.2)	21.2 (27.8)	1.9 (26.7)	−14.1 (15.5)	−1.9 (36.4)	−4.1 (28.2)	
**SBJ15**	9								
ERD/ERS BL1		−18.9 (29.6)	−25.6 (34.9)	−14.8 (30.9)	−35.6 (25.1)	−19.7 (20.1)	−10.1 (19.6)	−20.8 (28.2)	
ERD/ERS BL2		−4.2 (37.2)	−24.5 (30.4)	−8.9 (29.1)	−25.2 (33.5)	−16.0 (21.2)	−13.8 (24.3)	−30.2 (39.6)	
**SBJ16**	7								
ERD/ERS BL1		−25.8 (19.3)	−0.2 (35.8)	−16.8 (40.6)	−9.0 (23.5)	−18.3 (57.1)	−35.5 (17.9)	−42.6 (19.9)	
ERD/ERS BL2		−25.9 (17.8)	−9.3 (19.2)	−15.4 (28.6)	−9.8 (20.9)	−11.0 (36.7)	−27.2 (19.0)	−16.5 (20.8)	

**Table 3:** The table shows, for each subject of the Group 1, the number of AO epochs analyzed, AO ERD/ERS values (median (IQR)) for each channel cluster and for the two baselines (BL1, BL2), the results of the paired Wilcoxon test comparing AO ERD/ERS computed with BL1 and BL2 (FCR adjusted *p*-value), the baseline preference. * denotes the significant mu-rhythm desynchronization (One simple Wilcoxon signed rank test, FCR adjusted *p*-value < 0.050).

	N. AO Epochs	F_Left	F_Right	C_Left	C_Right	P_Left	P_Right	O	BL Preference
**GROUP 1**									
**SBJ2**	18								
ERD/ERS BL1		−21.1 (28.4)	3.7 (39.2)	−3.7 (24.3)	−9.6 (28.4)	−11.1 (18.0)	−17.8 (22.8)	−15.5 (30.1)	
ERD/ERS BL2		−5.3 (39.7)	2.8 (49.8)	5.1 (38.2)	−8.0 (27.0)	−11.6 (27.1)	17.2 (30.7)	−10.5 (53.2)	
**SBJ3**	25								
ERD/ERS BL1		−39.2 (15.3) *	−45.7 (10.3) *	−36.7 (18.8) *	−36.2 (19.9) *	−45.6 (16.1) *	−41.3 (15.3) *	−25.0 (18.9) *	
ERD/ERS BL2		−23.6 (19.2) *	−32.3 (12.8) *	−24.4 (22.1) *	−25.3 (23.1) *	−34.3 (19.8) *	−29.7 (18.5) *	−12.2 (22.1) *	
Wilcoxon test adj *p*		**<0.001**	**<0.001**	**<0.001**	**<0.001**	**<0.001**	**<0.001**	**<0.001**	BL1
**SBJ5**	19								
ERD/ERS BL1		−2.9 (53.0)	−17.3 (45.0)	−10.2 (43.0)	−7.1 (53.5)	−15.7 (41.2)	−24.1 (19.9) *	−7.7 (24.8)	
ERD/ERS BL2		−17.4 (18.5) *	−35.8 (15.4) *	−11.0 (22.3) *	−30.5 (21.5) *	−35.2 (17.9) *	−34.8 (11.4) *	−22.6 (13.9) *	
Wilcoxon test adj *p*		0.232	0.068	>0.999	0.132	**0.007**	0.132	**0.012**	BL2
**SBJ6**	23								
ERD/ERS BL1		−25.0 (34.6) *	−17.3 (20.9) *	−10.2 (35.6)	−23.1 (17.0) *	−21.3 (33.1) *	−15.9 (16.6) *	−8.0 (27.7)	
ERD/ERS BL2		6.9 (66.2)	−7.1 (41.2)	2.3 (28.8)	10.3 (45.0)	−9.9 (41.1)	−4.6 (34.8)	−1.4 (35.5)	
Wilcoxon test adj *p*		**<0.001**	**0.008**	0.642	**<0.001**	**0.008**	**0.008**	>0.999	BL1
**SBJ7**	15								
ERD/ERS BL1		−3.7 (40.4) *	−3.7 (18.9) *	−6.1 (51.9)	−11.7 (32.4) *	−20.9 (25.2) *	−16.4 (46.6) *	−13.8 (18.7)	
ERD/ERS BL2		11.2 (46.7)	13.2 (22.2)	9.2 (60.4)	2.7 (38.8)	−3.3 (31.0)	2.1 (57.0)	−8.6 (19.6)	
Wilcoxon test adj *p*		**<0.001**	**<0.001**	**<0.001**	**<0.001**	**<0.001**	**<0.001**	**<0.001**	BL1
**SBJ8**	16								
ERD/ERS BL1		−15.0 (22.8) *	−13.1 (32.3)	−29.6 (29.9) *	−15.0 (21.9)	−27.2 (22.1) *	−17.0 (19.1) *	−2.9 (30.8)	
ERD/ERS BL2		9.9 (38.6)	−24.4 (21.6) *	−0.5 (32.6)	−7.1 (23.5)	−20.1 (20.8) *	2.3 (17.8)	8.1 (40.3)	
Wilcoxon test adj *p*		**0.004**	0.772	**0.004**	0.845	**0.004**	**0.004**	**0.035**	BL1
**SBJ9**	14								
ERD/ERS BL1		0.4 (26.7)	−9.4 (33.0)	6.9 (23.2)	−9.3 (33.2)	−20.4 (32.6)	10.0 (16.4)	−3.8 (19.8)	
ERD/ERS BL2		−18.2 (28.3) *	−31.1 (31.7) *	−11.0 (15.0)	−22.4 (14.1) *	−33.2 (17.1) *	−19.4 (38.7) *	−9.5 (25.2)	
Wilcoxon test adj *p*		0.111	0.111	0.463	0.111	0.463	**0.002**	0.770	BL2
**SBJ11**	22								
ERD/ERS BL1		−18.6 (17.8) *	−28.4 (22.6) *	−8.7 (38.8)	−15.9 (42.7)	−27.2 (22.2) *	−12.4 (31.5) *	−14.7 (28.0) *	
ERD/ERS BL2		−11.9 (24.1)	−8.8 (36.6)	−18.1 (32.1)	2.7 (43.1)	−1.0 (28.0)	−14.8 (24.1)	0.1 (20.8)	
Wilcoxon test adj *p*		0.954	0.072	>0.999	0.072	**0.018**	>0.999	0.178	BL1
**SBJ12**	16								
ERD/ERS BL1		−23.6 (38.0)	−7.5 (13.3)	−3.8 (31.8)	−11.0 (20.3)	−19.7 (16.7)	−26.2 (27.2)	−2.5 (19.7)	
ERD/ERS BL2		−29.2 (34.7) *	−23.5 (10.8) *	−9.4 (29.1)	−18.7 (19.3) *	−30.7 (14.1) *	−35.8 (24.2) *	−23.7 (15.3) *	
Wilcoxon test adj *p*		**0.001**	**0.001**	**0.001**	**0.001**	**0.001**	**0.001**	**0.001**	BL2
**SBJ13**	19								
ERD/ERS BL1		−20.3 (16.3) *	−30.5 (37.0) *	−5.0 (37.5)	−3.3 (30.6)	−19.8 (30.7) *	−18.5 (26.6) *	−4.2 (23.3)	
ERD/ERS BL2		−10.4 (35.1)	−10.9 (28.7)	0.8 (27.7)	−8.4 (36.4)	4.3 (29.5)	−12.0 (27.4)	6.8 (31.4)	
Wilcoxon test adj *p*		0.2123	**0.012**	>0.999	0.839	**0.003**	**0.012**	**0.012**	BL1
**SBJ14**	20								
ERD/ERS BL1		−13.3 (14.8)	−12.3 (15.2)	−7.8 (24.1)	−7.8 (27.1)	−4.8 (25.7)	−10.8 (20.9)	−7.0 (17.3)	
ERD/ERS BL2		−6.3 (16.0)	−4.5 (16.5)	0.4 (26.3)	−5.5 (28.0)	0.6 (27.1)	−5.4 (22.0)	10.4 (20.5)	
**SBJ17**	5								
ERD/ERS BL1		−15.8(43.3)	−12.5 (32.4)	−19.2 (29.8)	11.3 (26.8)	−11.2 (42.2)	−24.3 (27.8)	−6.4 (26.1)	
ERD/ERS BL2		−27.3 (38.1)	−24.2 (29.2)	−29.0 (26.9)	−2.8 (23.5)	−16.1 (40.1)	−29.0 (25.2)	−0.6 (27.5)	
**SBJ18**	9								
ERD/ERS BL1		30.8 (29.5)	14.0 (55.1)	−14.5 (18.0)	−4.2 (47.1)	−1.5 (4.5)	−14.0 (39.1)	−13.6 (11.1)	
ERD/ERS BL2		26.3 (44.8)	11.7 (47.6)	18.2 (24.4)	17.7 (54.3)	5.4 (21.5)	23.7 (45.7)	0.4 (33.5)	
**SBJ19**	18								
ERD/ERS BL1		−19.4 (32.1) *	−16.9 (24.7) *	−19.1 (22.4) *	−22.3 (12.1) *	−24.0 (24.4) *	−15.6 (19.9) *	−15.6 (20.7)	
ERD/ERS BL2		−1.8 (44.6)	−5.4 (35.5)	10.2 (36.2)	−3.4 (12.7)	3.2 (26.9)	−3.4 (24.9)	−10.4 (34.8)	
Wilcoxon test adj *p*		**0.022**	**0.032**	**0.001**	**0.001**	**0.001**	**0.001**	**0.022**	BL1

**Table 4:** Summary of the main findings of the present study.

**Study Evidence**
The majority of the toddlers (73.7%) showed mu-rhythm desynchronization, as reported in the adult literature [22].
A low number of toddlers (26.3%) did not desynchronize the mu-rhythm in response to the task, as reported in the adult literature [22].
Only 47.4% of the subjects showed significant mu-ERD with both the baselines tested.
The majority of the mu-rhythm-suppressive subjects (92.8%) showed a baseline preference, as reported in the adult literature [22].
The observation of a static image (BL1) resulted in being the best baseline in our sample; this is in contrast with [22], where a common preferred baseline was not identified.
**Suggestions for Future Studies**
Verify the presence of a mu-rhythm peak in the single subjects’ baseline spectra.
Perform single-subject analysis and exclude from group analysis the subjects that are not sensitive to a specific type of baseline.
Include in the experimental protocol more than one baseline condition.
Be careful in comparing the results of studies that adopt different baselines.

## Data Availability

The dataset generated for this study is freely downloadable from Zenoodo.org (DOI: 10.5281/zenodo.5145887).
